# Complete Vascular Replacement of the Infrarenal Inferior Vena Cava and Abdominal Aorta during Post-Chemotherapy Retroperitoneal Lymph Node Dissection for a Non-Seminomatous Germ Cell Tumor

**DOI:** 10.3390/curroncol30060412

**Published:** 2023-06-04

**Authors:** Konstantinos Evmorfopoulos, Georgios Chasiotis, Alexandros Barbatis, Ioannis Zachos, George Kouvelos, Metaxia Bareka, Panagiotis J. Vlachostergios, Eleni Arnaoutoglou, Vassilios Tzortzis, Miltiadis Matsagkas

**Affiliations:** 1 Department of Urology, School of Health Sciences, Faculty of Medicine, University of Thessaly, University Hospital of Larissa, 41110 Larissa, Greece; 2 Department of Vascular Surgery, School of Health Sciences, Faculty of Medicine, University of Thessaly, University Hospital of Larissa, 41110 Larissa, Greece; 3 Department of Anesthesiology, School of Health Sciences, Faculty of Medicine, University of Thessaly, University Hospital of Larissa, 41110 Larissa, Greece; 4 Department of Medical Oncology, IASO Thessalias Hospital, 41500 Larissa, Greece; 5 Department of Medicine, Division of Hematology and Medical Oncology, Weill Cornell Medicine, New York, NY 10065, USA

**Keywords:** testicular germ cell tumors, retroperitoneal lymph node dissection, vascular reconstruction, vena cava, aorta

## Abstract

Testicular germ cell tumors (TGCTs) are the leading cause of cancer-related death in males between the ages of 20 and 40. In the advanced stages, the combination of cisplatin-based chemotherapy and surgical excision of the remaining tumor can cure many of these patients. Vascular procedures may be required during retroperitoneal lymph node dissection (RPLND) in order to achieve the complete excision of all residual retroperitoneal masses. Careful assessment of pre-operative imaging and the identification of patients who could benefit from additional procedures are important for minimizing peri- and postoperative complications. We report on a case of a 27-year-old patient with non-seminomatous TGCT, who successfully underwent post-chemotherapy RPLND with additional infrarenal inferior vena cava (IVC) and complete abdominal aorta replacement using synthetic grafts.

## 1. Introduction

Testicular cancer is the most common cancer among young men between 20 and 40 years of age, and even though it accounts for <1% of all tumors in men, its incidence is gradually rising [1,2]. Over the last few decades, germ cell tumors (GCTs) have emerged as a model for curable neoplasms. The cornerstone of treatment for advanced testicular germ cell tumors (TGCT), particularly those that are non-seminomatous, is cisplatin-based chemotherapy followed by the surgical removal of residual masses in the retroperitoneum with post-chemotherapy retroperitoneal lymph node dissection (PC-RPLND). This procedure achieves accurate pathological re-staging and therapeutic benefits by removing any remaining teratoma or cancer. The current multimodality treatments have resulted in a 10-year testis-cancer-specific survival rate of more than 95% [3]. The success of treatment depends greatly on the timely and thorough administration of both surgery and chemotherapy. Despite the effectiveness of platinum-based chemotherapy, approximately 25% of patients with stage II–III TCGT still have a residual retroperitoneal mass following the completion of chemotherapy. In various studies, this percentage, depending on the clinical stage, ranges between 10 and 40% for advanced and metastatic disease [4,5,6]. When any residual lymph node mass exceeds 1 cm after chemotherapy, it should be considered for surgical removal via retroperitoneal lymph node dissection (RPLND) [7]. Persistent lymph nodes often contain teratoma in 30–40% of patients and viable cancer in 10–20% of patients [8]. The aim of surgery is to remove all lymph nodes within the retroperitoneum around the great vessels, below the renal vessels, between the ureters, and above the bifurcation of the iliac vessels during a full bilateral retroperitoneal lymphadenectomy (pcRPLND).

pcRPLND can be a difficult surgical procedure due to the complex anatomy of the retroperitoneal space. It requires expert surgeons who are well-versed in retroperitoneal anatomy and capable of performing surgical techniques on the vascular and intestinal structures [9,10,11]. Compared to the primary RPLND procedure, pcRPLND is associated with higher morbidity rates, with the reported complication rates ranging from 1.2% to 23.3% [12]. Moreover, due to post-chemotherapy distortion of the tissue planes and infiltration of the tumor into adjacent structures, such as the duodenum, kidneys, ureters, aorta, and inferior vena cava (IVC), adjunctive procedures during RPLND may be required in up to one-third of cases to ensure the complete resection of residual masses. Nephrectomy is the most commonly performed additional procedure during pcRPLND, employed in approximately 5–19% of cases [13], while additional vascular surgical procedures are required less frequently, in approximately 6% of patients [14,15].

Donohue first described the split and roll technique over the aorta and vena cava, dividing the lumbar vessels in order to easily retract the great vessels and safely resect the retroperitoneal tumors during RPLND [16]. However, large tumors that adhere to the IVC and aorta may prevent complete resection from the great vessels, and therefore, IVC or aortic resection and replacement are indicated. In this setting, the infrarenal vena cava is predominantly involved, while less frequently, the aorta may be affected [17].

Herein, we report a case of a 27-year-old patient with a residual retroperitoneal mass after chemotherapy for non-seminomatous TGCT, who underwent pcRPLND with complete replacement of the infrarenal inferior vena cava (IVC) and abdominal aorta using synthetic grafts. We also review relevant literature on clinical and anatomical characteristics that could help to predict the need for major vascular surgery in these patients.

## 2. Case Presentation

A 27-year-old patient was initially diagnosed with testicular cancer after experiencing severe scrotal pain radiating to his back. On physical examination, a painless mass was palpated in the right testicle and confirmed on scrotal ultrasound, measuring 7.5 cm. A right orchidectomy was immediately scheduled and performed. Histopathological diagnosis of the resected mass was consistent with a non-seminomatous yolk sack tumor (95%) with a minor component of teratoma (5%).

Computed tomography (CT) of the chest, abdomen, and pelvis (CAP) revealed a retroperitoneal lymph node mass measuring 14 × 13 × 8.4 cm^3^ and bilateral pulmonary metastatic lesions. Serum marker assessment disclosed a significantly elevated AFP of 1620 ng/mL with a β-hCG of 573 mIU/mL and an LDH level of 1190 IU/L. The patient was initiated on platinum-based chemotherapy for stage IIIB intermediate-risk disease and received four cycles of a standard bleomycin, etoposide, and cisplatin (BEP) regimen. Reimaging with CT CAP after the completion of systemic therapy showed a complete response of the pulmonary metastatic sites but no significant size reduction in the retroperitoneal mass. Postoperative serum marker assessment was consistent with the normalization of AFP at 33.4 ng/mL, as well as β-hCG (0.1 mIU/mL) and decreased levels of LDH at 402 IU/L.

During preoperative assessment, the patient underwent CT angiography, which showed infiltration and compression of the inferior vena cava (IVC), as well as encasement of the abdominal aorta (AA) and the proximal part of both iliac arteries. Complete resection of the total infrarenal length of both major vessels up to the mid-iliac arteries and veins was discussed and thoroughly planned with the Vascular Surgery and Anesthesiology teams during the tumor board review of the case. Finally, the patient underwent RPLND with total replacement of the IVC and AA with synthetic grafts.

**Figure 1:**
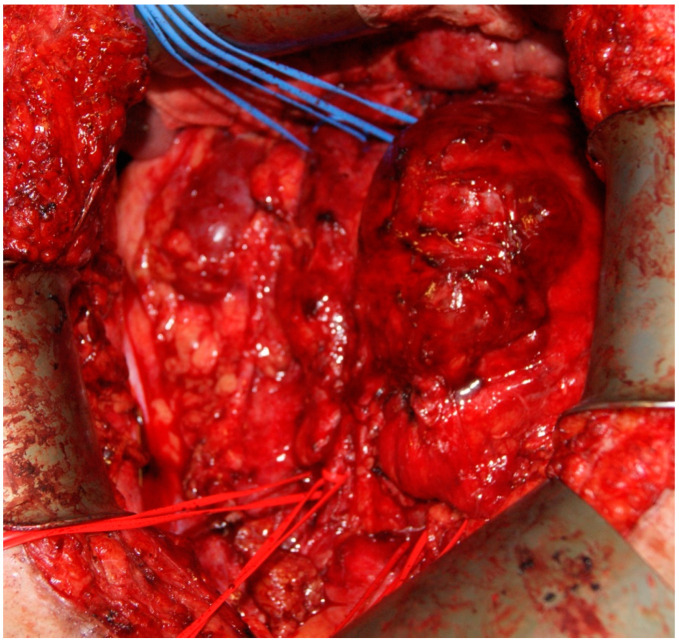
Intraoperative image showing the exposure of the retroperitoneal mass alongside the IVC and aorta, as well as the exposure of the proximal part of both iliac arteries and veins.

Intraoperatively, careful mobilization of the retroperitoneal mass, IVC, and abdominal aorta from the renal vessels to common iliac vessels was performed (Figure 1).

**Figure 2:**
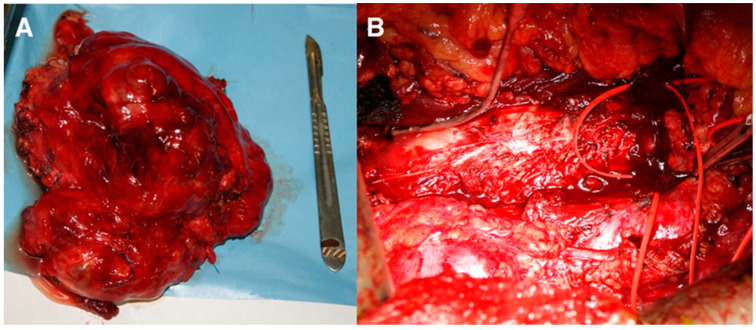
Intraoperative image revealing (**A**) the retroperitoneal mass specimen and (**B**) the retroperitoneal area after the resection of the mass.

Vascular clamps were placed inferior to the renal arteries and veins and bilaterally at the bifurcation of the common iliac veins and arteries. Complete resection of the retroperitoneal mass alongside the IVC and aorta, as well as the proximal part of both iliac arteries and veins, was achieved (Figure 2). An endovascular thrombus in the IVC was also recognized.

**Figure 3:**
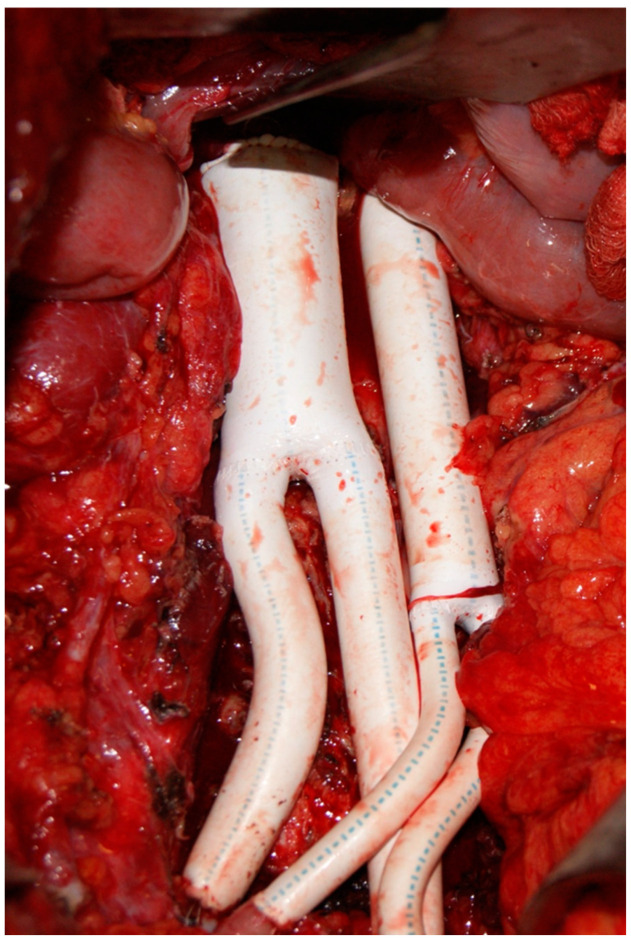
Intraoperative image depicting the total reconstruction of the abdominal aorta and inferior vena cava using synthetic grafts.

Subsequently, a replacement of the aorta and the common iliac arteries using a bifurcated 16 × 8 mm PTFE graft (Gore, AZ, USA) was performed with end-to-end anastomoses and the arterial blood flow was restored. A successful restoration of the veins was also performed using a bifurcated 24 × 12 mm PTFE graft (Gore, AZ, USA) (Figure 3).

**Figure 4:**
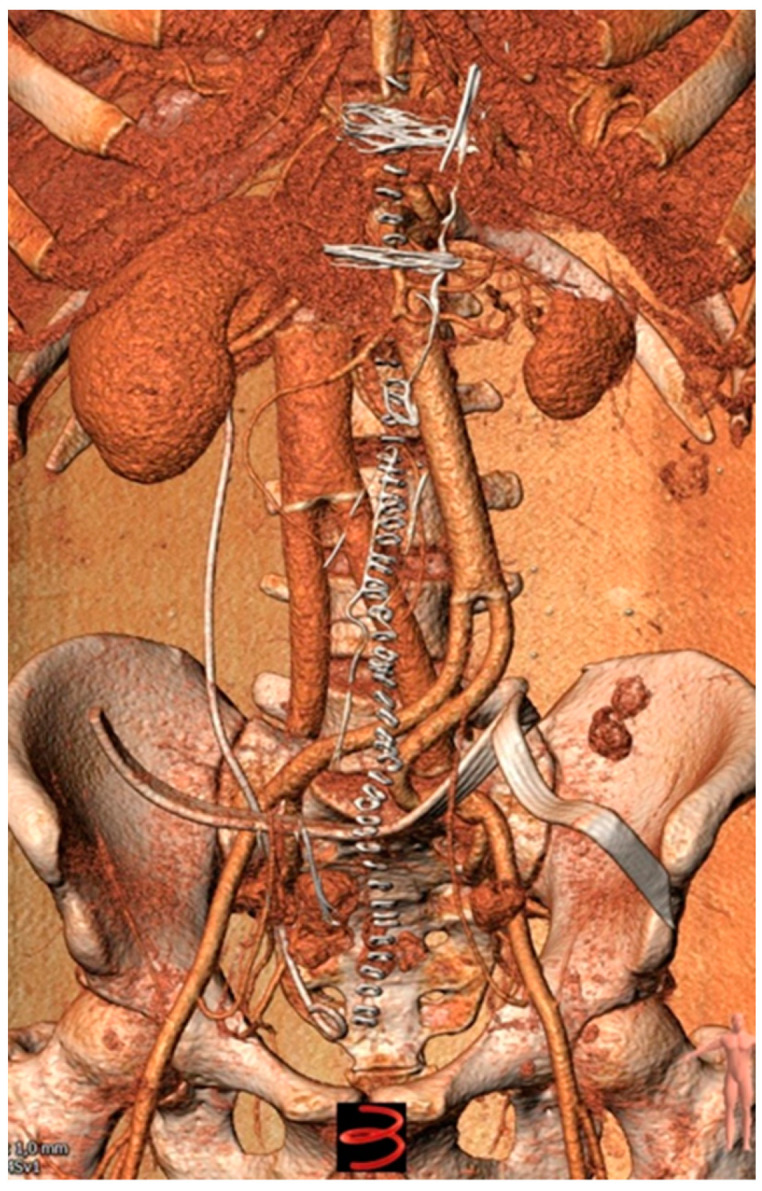
Computed tomography angiogram showing excellent patency of both synthetic grafts.

A tissue-traction-related injury to the right ureter was treated with the placement of a double J-stent end-to-end anastomosis. During the entire procedure, the patient was transfused with six units of packed red blood cells but remained hemodynamically stable and was transferred to the intensive care unit (ICU) postoperatively for monitoring. Prophylactic anticoagulation with enoxaparin 8.000 anti-Xa IU twice daily was started to reduce the thrombotic risk in the reconstructed inferior vena cava system. Post-op CT angiography did not disclose any complications, while revealing the full patency of both vascular grafts (Figure 4).

The hospital course was complicated by prolonged lymphatic leakage from the draining tube for an additional six-day period after discharge (corresponding to Grade I severity according to Clavien–Dindo classification). Moreover, a subcutaneous hematoma at the incision site required drainage after bedside removal of the surgical clips (Grade II complication). The patient was cleared for discharge by both Urology and Vascular Surgery teams on post-op day 12, starting rivaroxaban 20 mg once daily for 6 months to maintain the patency of the venous graft. The patient was re-evaluated 30 days postoperatively with serum biomarkers showing a further decrease in AFP within the normal range at 1.62 ng/mL, while adequate patency of the vascular grafts was confirmed via color Duplex ultrasonography. Histopathological evaluation of the resected masses showed the presence of viable teratoma with minor components of yolk sac tumor in the retroperitoneal mass, and therefore, the patient was started on second-line chemotherapy with paclitaxel-ifosfamide-cisplatin (TIP).

## 3. Discussion

pcRPLND is an integral part of the treatment algorithm for patients with advanced non-seminomatous TGCTs. It is a complex surgical procedure, and occasionally, patients may require additional procedures, including vascular surgical resections, usually involving the IVC. NSGCT residuals are often found attached to the vascular wall, but in some cases, they may be located within the lumen of great vessels. After chemotherapy, the incidence of intraluminal thrombi in RPLND can be as high as 5.8% [17]. When these thrombi occur unexpectedly, they may result in serious and life-threatening complications during surgery [17].

If lesions cannot be separated from the great vessels or are located inside the lumen, vascular surgery techniques must be employed. These techniques may include cavotomy and thrombectomy, partial or complete cavectomy, aortic resection, or complete vascular replacement. Caldarelli et al. [18] suggested that tumor residuals involving less than half the circumference of the vena cava may be managed through primary suture or patch techniques, while lesions involving more than 50% of the circumference require complete resection. Ligation or complete resection of the infrarenal IVC is sometimes performed without the need for synthetic graft reconstruction due to the presence of collateral vasculature. On rare occasions, patients may develop distal venous thrombosis, resulting in limb edema and even venous ulceration in the long term [13,15].

IVC reconstruction may help to prevent complications that can develop after IVC ligation, and certain clinical and intraoperative factors may play key roles in the decision on IVC reconstruction. According to Ehrlich et al. [15], there are four key considerations when addressing vena caval involvement during RPLND. First, it is important to completely remove any vena caval thrombus present during surgery, as it may contain mature teratoma or viable cancer [17]. This is typically accomplished through cavotomy, followed by primary repair of the incision without the need for vena caval reconstruction. Second, when the tumor is adhered to or penetrates part of the caval wall, graft-patch repair of the IVC is preferred over partial excision and primary suture, as it prevents significant narrowing. Third, if the entire circumference of the previously patent IVC is encased by the tumor or the IVC is completely occluded, causing significant symptoms, total reconstruction of the vein using a prosthetic graft is preferred. Finally, in patients with long-standing venous obstruction, where the IVC cannot be separated from the tumoral mass and there are significant venous collaterals, the IVC should be resected and ligated below the renal veins beyond the level of disease involvement. Since the IVC, in these cases, has little hemodynamic significance, its reconstruction is likely to be unnecessary.

Regarding aortic involvement, infiltration of the aorta by the residual mass is an absolute indication for complete resection and replacement with a synthetic graft. Aortic resection may be indispensable for achieving an R0 resection, in addition to the fact that peeling the tumor from the aorta may leave the aorta with a thin wall [14,19]. Several studies have demonstrated that patients who undergo complex pcRPLND typically exhibit significant histopathologic findings such as teratoma, vital cancer, or metastases in the majority of vascular specimens, whereas those who undergo standard pcRPLND show necrosis or fibrosis in approximately 25–33% of vascular specimens [15,17]. Thus, complete resection of the aorta for complete tumor clearance results in better oncological outcomes [12,14]. In the described case, the involvement of both vascular structures was visible on pre-operative imaging, facilitating pre-op planning of the tumor’s resection *en bloc* with the aorta and IVC to achieve an R0 resection. A vascular reconstruction was then successfully performed using synthetic PTFE grafts, followed by long-term anticoagulation to sustain the patency of the venous graft.

Even though laparotomy is the standard approach for patients with excessive retroperitoneal residuals and cases where vascular replacement is needed, recent studies have demonstrated advances in laparoscopic surgical techniques that have made vascular reconstruction during laparoscopic RPLND feasible. Aufderklamm et al. [20] studied 19 patients with NSGCT who underwent laparoscopic pcRPLND with complete intracorporeal IVC reconstruction. All men received bilateral laparoscopic RPLND, while infiltration of the great vessels was confirmed intraoperatively. Cavotomy was performed using laparoscopic bulldog and Satinski clamps, while the vessel wall was reconstructed using vascular surgery technics. There was no conversion to open surgery, while no distant or in-field relapse was observed during the follow-up. However, in this setting, the surgeon must be familiar with these laparoscopic techniques in order to achieve excellent oncological results, and therefore, laparotomy remains the gold standard of treatment in these patients.

There is currently an unmet need to develop and prospectively validate predictive markers which could help to identify patients who are likely to need vascular surgery during RPLND. According to the German Testicular Cancer Study Group, the risk factors for vascular involvement include intermediate- or poor-risk germ cell tumors, according to the International Germ Cell Cancer Collaborative Group risk classification, and the presence of residual masses that exceed 5 cm after chemotherapy [11]. Patients with these criteria presented a 4.61-fold increased risk of vascular involvement, and every fifth patient exhibiting one of these criteria needed some type of vascular procedure. A more recent study of 97 patients highlighted the degree of great vessel circumferential involvement, including IVC > 135° and AA > 330° as independent predictive indicators of the need for resection or reconstruction, with high negative predictive values (92% and 97%, respectively) [19]. In addition to a poor IGCCCG score, other studies recently described metrics that could also serve as predictive tools for the need for major vascular surgical procedures, including the aorta– and cava–tumor contact angle (≥64° and ≥98°, respectively) [21].

The long-term sequelae of IVC resection during RPLND after a median follow-up of over 7 years were reassuring, without long-term disability in three-quarters of patients [22]. In addition to multidisciplinary collaboration and management, the centralization of pcRPLND in high-volume centers is key to minimizing complications and ensuring no significant difference in outcomes despite higher-complexity cases. This finding was reported by several groups, including the largest UK series, consisting of 178 patients, among whom the vascular reconstruction rate was 6% [23]. Additionally, an Italian high-volume center disclosed that two-thirds of patients who underwent IVC surgery were alive without evidence of disease or major complications, except for one with significant lymphedema, after a follow-up of 43–207 months [24]. Bulky retroperitoneal masses > 10 cm, as part of growing teratoma syndrome, have also been reported to require aortic section with anastomosis, aortic grafting, IVC resection, or all of these procedures [25]. Despite presence of complications in one-quarter of the patients, the oncological outcomes were satisfactory, with only 1 out of 12 patients experiencing paraortic recurrence after a median follow-up of 5 years in a French cohort [25]. Overall, caval or/and aortic involvement indicates a higher-risk subgroup of patients and should be addressed in experienced medical centers due to its moderate long-term morbidity. Robotic RPLND pre- or post-chemotherapy is currently gaining ground as a novel surgical approach to minimize operating times and blood loss. The reported rate of additional vascular procedures is approximately 7%; however, long-term follow-up to assess whether the oncological outcomes are comparable to the those of the open approach is currently lacking [26].

## 4. Conclusions

pcRPLND encompasses complex surgical procedures with potential vascular involvement, depending on certain clinical (poor-risk ICCCGC classification) and anatomical parameters (large tumors, proximity, and the circumferential involvement of large vessels). Concomitant aorto-caval reconstruction, when necessary, as in our case, increases the complexity and concern regarding the patency of aorto-caval bypass grafts and the avoidance of lower extremity ischemia or edema. Therefore, adequate preoperative evaluation may help to identify those patients who will benefit from vascular reconstruction, while careful planning and execution of the procedure in high-volume centers of reference could minimize post-operative complications and improve oncological outcomes.

## Data Availability

The data presented in this study are available within the article.

## References

[1] Gilligan T., Lin D.W., Aggarwal R., Chism D., Cost N., Derweesh I.H., Emamekhoo H., Feldman D.R., Geynisman D.M., Hancock S.L. (2019). Testicular Cancer, Version 2.2020, NCCN Clinical Practice Guidelines in Oncology. J. Natl. Compr. Cancer Netw..

[2] Park J.S., Kim J., Elghiaty A., Ham W.S. (2018). Recent Global Trends in Testicular Cancer Incidence and Mortality. Medicine (Baltimore).

[3] Fosså S.D., Cvancarova M., Chen L., Allan A.L., Oldenburg J., Peterson D.R., Travis L.B. (2011). Adverse Prognostic Factors for Testicular Cancer-Specific Survival: A Population-Based Study of 27,948 Patients. J. Clin. Oncol..

[4] Lavery H.J., Bahnson R.R., Sharp D.S., Pohar K.S. (2009). Management of the residual post-chemotherapy retroperitoneal mass in germ cell tumors. Ther. Adv. Urol..

[5] Wells H., Hayes M.C., O’Brien T., Fowler S. (2017). Contemporary retroperitoneal lymph node dissection (RPLND) for testis cancer in the UK—A national study. BJU Int..

[6] Hendry W.F., Norman A.R., Dearnaley D.P., Fisher C., Nicholls J., Huddart R.A., Horwich A. (2002). Metastatic nonseminomatous germ cell tumors of the testis: Results of elective and salvage surgery for patients with residual retroperitoneal masses. Cancer.

[7] Heidenreich A., Pfister D., Witthuhn R., Thüer D., Albers P. (2009). Postchemotherapy Retroperitoneal Lymph Node Dissection in Advanced Testicular Cancer: Radical or Modified Template Resection. Eur. Urol..

[8] Honecker F., Aparicio J., Berney D., Beyer J., Bokemeyer C., Cathomas R., Clarke N., Cohn-Cedermark G., Daugaard G., Dieckmann K.-P. (2018). ESMO Consensus Conference on Testicular Germ Cell Cancer: Diagnosis, Treatment and Follow-Up. Ann. Oncol..

[9] Mosharafa A.A., Foster R.S., Koch M.O., Bihrle R., Donohue J.P. (2004). Complications of Post-Chemotherapy Retroperitoneal Lymph Node Dissection for Testis Cancer. J. Urol..

[10] Cary C., Masterson T.A., Bihrle R., Foster R.S. (2015). Contemporary Trends in Postchemotherapy Retroperitoneal Lymph Node Dissection: Additional Procedures and Perioperative Complications. Urol. Oncol..

[11] Winter C., Pfister D., Busch J., Bingöl C., Ranft U., Schrader M., Dieckmann K.-P., Heidenreich A., Albers P. (2012). Residual Tumor Size and IGCCCG Risk Classification Predict Additional Vascular Procedures in Patients with Germ Cell Tumors and Residual Tumor Resection: A Multicenter Analysis of the German Testicular Cancer Study Group. Eur. Urol..

[12] Heidenreich A., Haidl F., Paffenholz P., Pape C., Neumann U., Pfister D. (2017). Surgical Management of Complex Residual Masses Following Systemic Chemotherapy for Metastatic Testicular Germ Cell Tumours. Ann. Oncol..

[13] Djaladat H., Nichols C., Daneshmand S. (2012). Adjuvant Surgery in Testicular Cancer Patients Undergoing Postchemotherapy Retroperitoneal Lymph Node Dissection. Ann. Surg. Oncol..

[14] Beck S.D., Foster R.S., Bihrle R., Koch M.O., Wahle G.R., Donohue J.P. (2001). Aortic Replacement during Post-Chemotherapy Retroperitoneal Lymph Node Dissection. J. Urol..

[15] Ehrlich Y., Kedar D., Zelikovski A., Konichezky M., Baniel J. (2009). Vena Caval Reconstruction during Postchemotherapy Retroperitoneal Lymph Node Dissection for Metastatic Germ Cell Tumor. Urology.

[16] Donohue J.P. (1977). Retroperitoneal Lymphadenectomy: The Anterior Approach Including Bilateral Suprarenal-Hilar Dissection. Urol. Clin. N. Am..

[17] Johnston P., Beck S.D.W., Cheng L., Masterson T.A., Bihrle R., Kesler K., Foster R.S. (2013). Incidence, Histology and Management of Intraluminal Thrombus at Post-Chemotherapy Retroperitoneal Lymph Node Dissection. J. Urol..

[18] Caldarelli G., Minervini A., Guerra M., Bonari G., Caldarelli C., Minervini R. (2002). Prosthetic Replacement of the Inferior Vena Cava and the Iliofemoral Vein for Urologically Related Malignancies. BJU Int..

[19] Johnson S.C., Smith Z.L., Nottingham C., Schwen Z.R., Thomas S., Fishman E.K., Lee N.J., Pierorazio P.M., Eggener S.E. (2019). Clinical and Radiographic Predictors of Great Vessel Resection or Reconstruction During Retroperitoneal Lymph Node Dissection for Testicular Cancer. Urology.

[20] Aufderklamm S., Todenhöfer T., Hennenlotter J., Mischinger J., Böttge J., Rausch S., Halalsheh O., Stenzl A., Gakis G., Schwentner C. (2014). Postchemotherapy Laparoscopic Retroperitoneal Lymph Node Dissection for Nonseminomatous Germ Cell Tumors Infiltrating the Great Vessels. J. Endourol..

[21] Nini A., Boschheidgen M., Hiester A., Winter C., Antoch G., Schimmöller L., Albers P. (2022). Preoperative Clinical and Radiographic Predictors of Major Vascular Surgery in Patients with Testicular Cancer Undergoing Post-Chemotherapy Residual Tumor Resection (PC-RPLND). World J. Urol..

[22] Beck S.D., Lalka S.G. (1998). Long-term results after inferior vena caval resection during retroperitoneal lymphadenectomy for metastatic germ cell cancer. J. Vasc. Surg..

[23] Pearce A.K., Manson-Bahr D., Reid A., Huddart R., Mayer E., Nicol D.L. (2021). Outcomes of Postchemotherapy Retroperitoneal Lymph Node Dissection from a High-volume UK Centre Compared with a National Data Set. Eur. Urol. Open Sci..

[24] Tavolini I.M., Norcen M., Oliva G., Nigro F., Benedetto G., Mazzariol C., Bassi P. (2002). II coinvolgimento cavale nei tumori non seminomatosi del testicolo in stadio avanzato: Strategie chirurgiche e risultati a lungo termine [Caval involvement in advanced-stage non-seminoma testicular tumors: Surgical strategy and long-term results]. Arch. Ital. Urol. Androl..

[25] Stella M., Gandini A., Meeus P., Aleksic I., Flechon A., Cropet C., Droz J.P., Rivoire M. (2012). Retroperitoneal vascular surgery for the treatment of giant growing teratoma syndrome. Urology.

[26] Nason G.J., Kuhathaas K., Anson-Cartwright L., Jewett M.A.S., O’Malley M., Sweet J., Hansen A., Bedard P., Chung P., Hahn E. (2022). Robotic retroperitoneal lymph node dissection for primary and post-chemotherapy testis cancer. J. Robot. Surg..

